# First mammogram-detected cancers portend worse survival in young women diagnosed with breast cancer

**DOI:** 10.1007/s10549-025-07703-9

**Published:** 2025-04-25

**Authors:** Avia D. Wilkerson, Megan Obi, Corey Gentle, Wei Wei, Camila Ortega, Zahraa Al-Hilli

**Affiliations:** 1 Department of General Surgery, Digestive Disease and Surgery Institute, Cleveland Clinic, 9500 Euclid Avenue/A10 Cleveland, Cleveland, OH 44195 USA; 2 Department of Quantitative Health Sciences, Cleveland Clinic, Cleveland, OH USA; 3 Breast Center, Integrated Surgical Institute, Cleveland Clinic, Cleveland, United States

**Keywords:** Breast cancer, Screening, Disparities, Survival, Recurrence

## Abstract

**Purpose:**

Breast cancer (BC) screening guidelines for women ages 40–45 have differed across multiple organizations, resulting in variable ages of screening commencement among women in the US. We previously reported that delay in screening beyond age 40 increases risk for first mammogram cancer diagnoses. We hypothesize that first mammogram cancer detection may also diminish recurrence-free and overall survival (RFS, OS).

**Study design:**

This retrospective cohort study included 738 women diagnosed with BC from ages 40–45 years and treated within a single hospital system from 2010 to 2019. First mammogram cancers were defined as those with tissue diagnoses established within 3 months of baseline mammogram. RFS after surgery and OS after BC diagnosis were analyzed in patients diagnosed on first versus subsequent mammograms via the Kaplan–Meier method, with p-values generated by log rank tests. Cumulative incidences of local and distant recurrence were also assessed.

**Results:**

Of 738 women, 218 had first mammogram cancers while 520 were diagnosed on subsequent mammograms. Median follow-up was 72.2 months (0.5–155.8 months). At 5 and 10 years after diagnosis, OS was significantly worse in patients diagnosed on their first mammogram (0.88 [0.83–0.93] and 0.80 [0.73–0.87]) versus subsequent mammograms (0.95 [0.93–0.97] and 0.90 [0.86–0.93]), *p* = 0.003. Patients with first mammogram cancers also had inferior 5– and 10–years RFS rates (0.81 [0.71–0.88] and 0.74 [0.65–0.83] vs. 0.88 [0.85–0.92] and 0.77 [0.72–0.83]), *p* = 0.04.

**Conclusion:**

First mammogram cancers were associated with worse survival in our study cohort, reinforcing the importance of consistent guidelines for screening commencement at age 40.

## Introduction

Breast cancer (BC) is the most common cancer in women in both developed and developing countries [1, 2]. While incidence rates have slowly increased over the past several decades, mortality rates have steadily declined for women of most racial/ethnic groups. However, there remain unwavering outcome disparities, with worse 5-years relative survival noted amongst Black women and increased mortality noted amongst women diagnosed at ages younger than 50 years [3]. Screening enables the best opportunity for early detection and has been associated with significant reduction in morbidity and disease-specific mortality [4]. Studies have demonstrated that screening has resulted in a 41% reduction in the development of cancers which would otherwise be fatal within 10 years of diagnosis [4, 5].

Unfortunately, there has been poor consensus in optimal screening guidelines across women’s health, surgical breast, oncologic, and radiologic societies and organizations. The American Academy of Family Physicians (AAFP) [6] and American College of Physicians (ACP) [7] currently recommend commencing screening at 50 years of age whereas the American Cancer Society (ACS) [8] recommends starting at age 45. Most recently, the United States Preventative Service Task Force (USPSTF) updated their 2016 guidelines in favor of biennial screening starting at age 40 rather than 50 due to increasing data demonstrating increased incidence of invasive cancer in women 40–49 years old as well as evidence of significant reduction in lifetime risk of cancer-related deaths when screening commences at age 40 [9]. Nevertheless, this recommendation still differs from those of the American College of Obstetrics and Gynecologists (ACOG) [10], American College of Radiology (ACR) [11], American Society of Breast Surgeons (ASBrS) [12], and National Comprehensive Cancer Network (NCCN) [13] guidelines which recommend annual screening beginning at age 40.

Given variability in screening recommendations, further understanding of the clinical implications of delayed screening and significance of BC detection on first mammogram may inform changes in practice and prevention that foster more equitable reduction of cancer-related morbidity and mortality. Such advancement of knowledge could greatly improve persisting disparities in BC diagnosis and survival. In addition, many social determinants of health, including socioeconomic status, have been shown to compromise BC mortality [14]. Improving access to early screening serves as an essential tool to combat outcome disparities. Furthermore, earlier screening results in greater mortality reduction in younger populations, for whom prior studies have shown worse outcomes [15]. Our group previously sought to elucidate the frequency of first mammogram cancers amongst various racial groups and determined that delaying screening after age 40 resulted in increased risk of first mammogram cancer diagnoses across races, though particularly in Black women [16]. The present study seeks to expound on the long-term oncologic outcomes of women diagnosed with BC on their first mammogram. We hypothesize that cancers diagnosed on first mammogram portend worse recurrence-free survival (RFS) and overall survival (OS) than cancers diagnosed on subsequent mammograms.

## Methods

Institutional Review Board approval was obtained for this retrospective cohort study performed within a single hospital system. Inclusion criteria were women ages 40 to 45 diagnosed with BC who underwent oncologic treatment for BC between 2010 and 2019. Women diagnosed at ages younger than age 40, older than age 45, and those with known hereditary breast cancer mutations identified prior to diagnosis were excluded from this study.

In our initial study, age at diagnosis, self-reported race, tumor characteristics, genetic testing outcomes, clinical staging and radiographic data were collected via our institution’s tumor registry and electronic health record. Radiographic data included the dates of first mammogram, mammogram associated with cancer diagnosis, and whether symptoms were present when the patient presented for the mammogram associated with eventual cancer diagnosis. Patients were identified as ‘symptomatic’ at diagnosis if they presented with a palpable mass, skin changes, discharge, or focal breast pain. Screening and diagnostic mammograms were included in our review and analyses. We defined first mammogram cancers as those with tissue diagnoses established within 3 months of baseline mammogram. Subsequent mammogram cancers were defined as those diagnosed one or more years after baseline mammogram or interval cancers diagnosed upon presentation with symptoms less than one year after a negative screening mammogram. Diagnostic mammograms that were performed for further work-up of baseline screening mammograms, such as those assigned a Breast Imaging Reporting and Data System (BIRADS) score of 0, were included in the first mammogram cohort [16]. To investigate differences in survival between patients diagnosed on first versus subsequent mammograms, dates of last follow up, recurrence data, vital statuses, and dates of death, if applicable, were obtained from the same cohort of 738 women. RFS was defined as duration of patient survival following surgery without signs or symptoms of recurrence. OS was defined as duration of survival from date of diagnosis to date of last follow-up or date of death.

Cumulative incidence rates of local and distant recurrence were estimated and compared by Gray’s method. RFS and OS were estimated by Kaplan–Meier method, with p-values generated by log rank test. Multivariate Cox proportional hazard models were fit to assess the effect of demographic factors, clinical factors, and timing of mammographic detection cancer diagnoses on RFS and OS. A backwards elimination procedure was employed to establish the final multivariate model. Age of diagnosis and diagnosis on first versus subsequent mammograms were forced into the models to assess their effects. All analyses were performed on a complete-case basis. Tests were two-tailed and performed at a significance level of 0.05. Statistical analysis was executed using SAS Studio 3.7 (SAS Institute, Cary, NC) and R version 4.1 (R Foundation, Vienna, Austria).

## Results

Demographic and clinicopathologic characteristics of this cohort were published and can be referenced in our prior work. In summary, 610 (82.5%) self-identified as White, 82 (11.1%) as Black women, 21 (2.8%) as Asian/Pacific Islander, 13 (1.8%) as Hispanic/Latina, 3 (0.4%) as American Native American/Alaskan Native, and the remaining 1.2% of enrolled patients identified as multiracial or deferred identifying their race. The median age of diagnosis across all racial groups was 43.0. Of 738 women, 218 had first mammogram cancers while 520 were diagnosed on subsequent mammograms. Median follow-up was 72.2 months (range 0.5–155.8 months) [16].

### Recurrence-free survival

Details of recurrence events were available for 83% of the 738 women reviewed (Table 1). Overall, locoregional recurrences were rare, with only 16 (17% of all recurrence events) noted on follow up. Distant recurrences, however, were more frequent, with 78 (83% of all recurrence events) confirmed cases. Of women who developed distant recurrences, 52% had metastatic disease at multiple sites. Median RFS was not reached, with an overall 5 years RFS rate of 86% and 10 years rate of 76%. Five patients developed new breast cancers (1 ipsilateral; 4 contralateral). Of note, 23 women (3% of all patients) either presented with metastatic disease or developed signs or symptoms of metastatic disease less than 6 months after undergoing oncologic surgery.

**Table 1:** Recurrence statuses and nature of known recurrence events

Recurrence status	Number (*N*)	Percentage (%)
No recurrence	493	66.80
Known recurrence	94	12.73
Never disease-free	24	3.25
Unknown	127	17.21
*Nature of recurrence*		
*Locoregional, total*	16	17.02
Breast, chest wall	14	14.89
Regional lymph nodes	2	2.12
*Distant, total*	78	82.98
Bone	13	13.83
Liver	2	2.12
Brain/CNS	3	3.19
Lung	7	7.44
Multiple sites	49	52.12
Other	4	4.25

There were significant differences in distant recurrence rates in women with first versus subsequent mammogram cancer diagnoses, particularly at 5 years from date of surgery. The 5 and 10 years distant recurrence rates for women with first mammogram cancers were 16% and 18% compared to 8% and 14% in women diagnosed on subsequent mammograms (*p* = 0.03). Due to the low number of locoregional recurrences, there was insufficient power to detect a statistically significant difference in locoregional recurrence by timing of mammographic detection (Table 2).

**Table 2:** Cumulative incidence of local and distant recurrence

Endpoint	Cohort	Estimated 5 years rate	5 years confidence interval	Estimated 10 years rate	10 years confidence interval	*P*-value
Locoregional recurrence	Overall	0.019	[0.007, 0.030]	0.052	[0.022, 0.083]	
	First mammogram	0.006	[0.000, 0.018]	0.063	[0.000, 0.131]	0.880
	Subsequent mammogram	0.023	[0.008, 0.038]	0.049	[0.015, 0.082]	
Distant recurrence	Overall	0.105	[0.079, 0.131]	0.155	[0.117, 0.193]	
	First mammogram	0.161	[0.101, 0.221]	0.182	[0.117, 0.247]	0.033
	Subsequent mammogram	0.084	[0.056, 0.112]	0.144	[0.098, 0.190]	

**Fig. 1:**
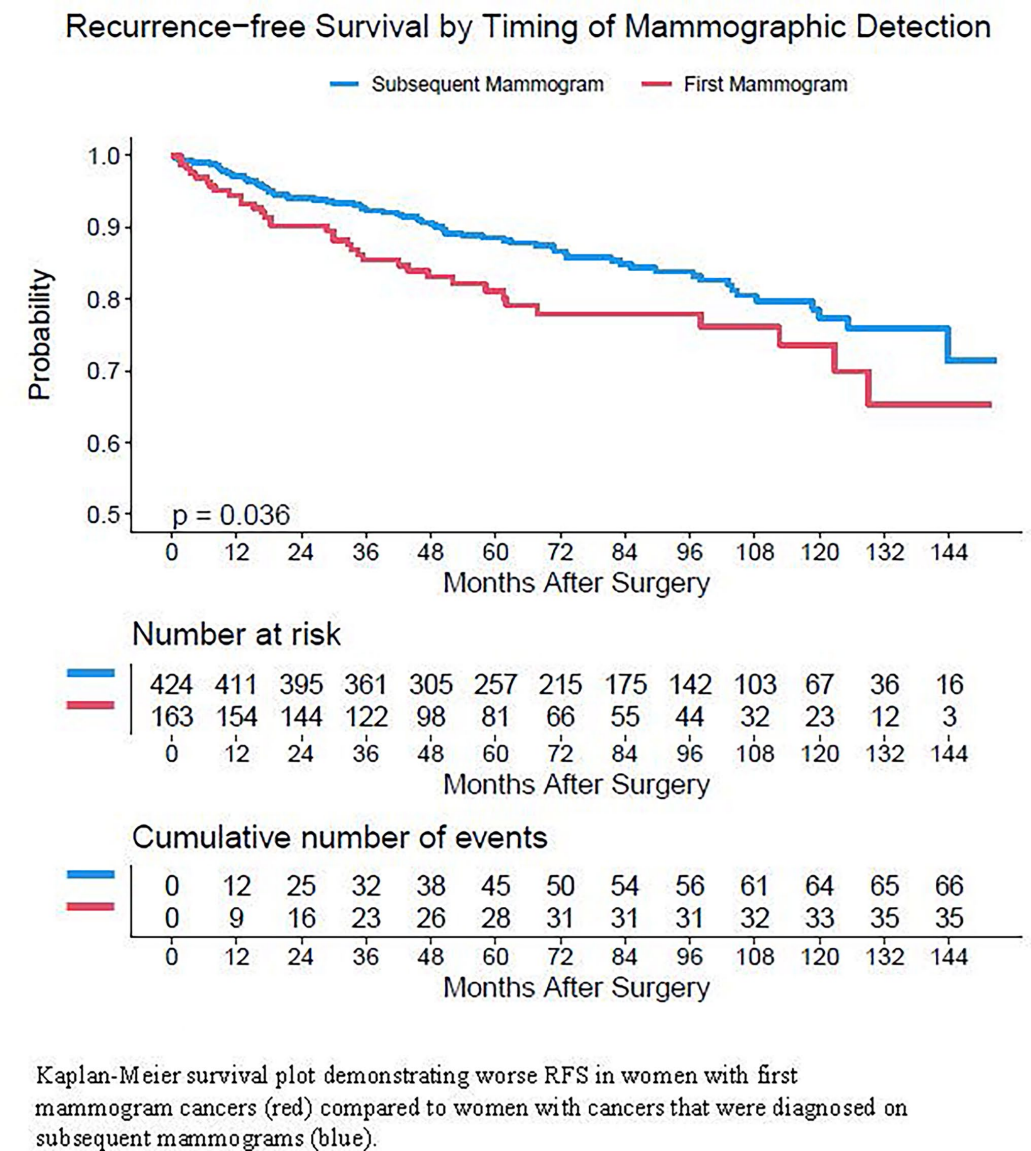
Kaplan–Meier survival plot for recurrence-free survival by timing of mammographic detection

Women with first mammogram cancers had significantly worse RFS than women diagnosed on subsequent mammograms (Fig. 1). As shown in Table 3, 5 and 10 years RFS rates in women with first mammogram cancers were 81% and 74% while those in women diagnosed on subsequent mammograms were 88% and 77% (*p* = 0.04). Though there was no statistically significant difference by race, there was a strong trend towards worse RFS in Black women compared with White women and women of other racial/ethnic groups (Table 3).

**Table 3:** Recurrence-free Survival rates by patient cohort

Cohort	Total N	Number of events	Rate at 5 years (95% CI^a^)	Rate at 10 years (95%CI)	*P*-value
All patients	587	101	0.86 (0.84, 0.89)	0.76 (0.72, 0.81)	
First Mammogram	163	35	0.81 (0.75, 0.88)	0.74 (0.65, 0.83)	0.04
Subsequent Mammogram	424	66	0.88 (0.85, 0.92)	0.77 (0.72, 0.83)	
White	490	81	0.87 (0.84, 0.90)	0.78 (0.73, 0.83)	0.17
Black	68	16	0.81 (0.71, 0.91)	0.66 (0.49, 0.87)	
Other groups	29	4	0.89 (0.78, 1)	0.71 (0.45, 1)	

^a^*CI* Confidence interval

A multivariate cox regression model for RFS revealed that first mammogram cancers were not significantly associated with RFS after adjusting for other factors (HR 1.46, *p* = 0.09). However, factors associated with disease burden at diagnosis were significantly associated with RFS, such as the presence of symptoms during the mammogram on which cancer was diagnosed (hazard ratio [HR] 1.78, *p* = 0.02) and clinical stage, particularly stage II (HR 2.73, *p* = 0.003), stage III (HR 6.65, *p* < 0.0001), and stage IV (HR 5.01, *p* = 0.01) disease. There was no significant association between age at diagnosis and RFS (Table 4).

**Table 4:** Multivariate analysis of factors associated with RFS

Factor	Comparison	Hazard ratio (95% confidence interval)	*P*-value
First mammogram diagnosis	Yes vs. No	1.461 (0.95, 2.247)	0.09
Symptomatic at diagnosis	Yes vs. No	1.779 (1.097, 2.884)	0.02
Clinical stage	Stage II vs. Stage I	2.734 (1.596, 4.685)	0.0003
	Stage III vs. Stage I	6.646 (3.637, 12.147)	< 0.0001
	Stage IV vs. Stage I	5.007 (1.465, 17.119)	0.01
	DCIS vs. I	0.692 (0.276, 1.737)	0.43
Age at diagnosis	1 year increase	1.093 (0.968, 1.235)	0.16

### Overall Survival

**Fig. 2:**
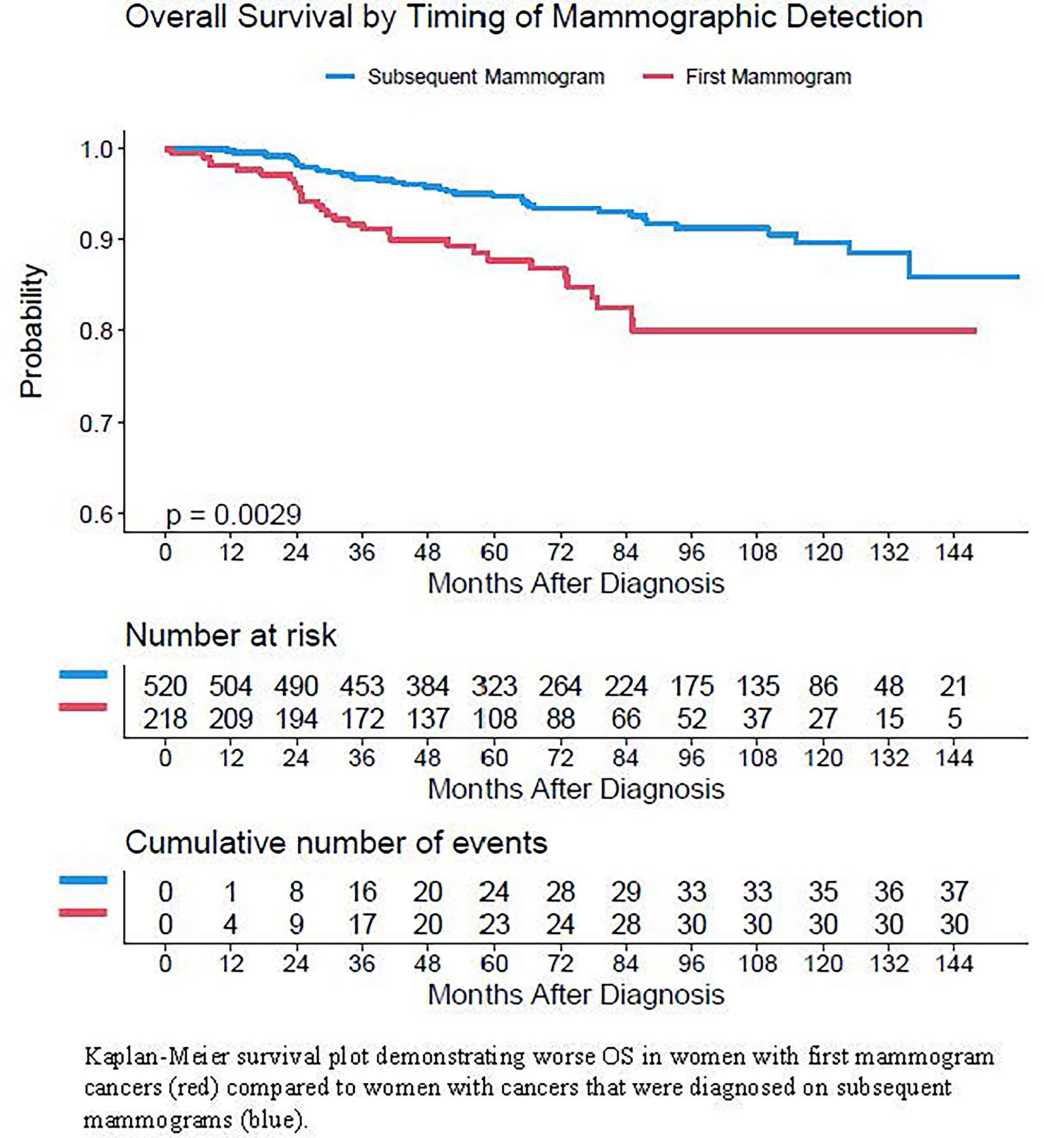
Kaplan–Meier survival plot for overall survival by timing of mammographic detection

The overall 5 years survival rate in this patient cohort was 93% and 10 years survival rate was 87% (Table 5). OS was significantly poorer in women diagnosed on their first versus subsequent mammogram on Kaplan–Meier analysis (Fig. 2). In women with first mammogram cancer diagnoses, OS at 5 and 10 years were 88% and 68% compared to 95% and 90% in women diagnosed on subsequent mammograms (*p* = 0.003) (Table 4). At both time points, there existed racial disparities in OS. Black women experienced significantly worse 5 years (86%) and 10 years (68%) overall survival than White women (93% and 89%) and women of other racial/ethnic groups (97% and 97%) (*p* = 0.002).

**Table 5:** Overall survival rates by patient cohort

Cohort	Total *N*	Number of events	Rate at 5 years (95% CI^a^)	Rate at 10 years (95%CI)	*P*-value
All patients	738	68	0.93 (0.91, 0.95)	0.87 (0.84, 0.9)	
First Mammogram	218	31	0.88 (0.83, 0.93)	0.8 (0.73, 0.87)	0.003
Subsequent Mammogram	520	37	0.95 (0.93, 0.97)	0.90 (0.86, 0.93)	
White	610	52	0.93 (0.91, 0.95)	0.89 (0.85, 0.92)	0.002
Black	82	15	0.86 (0.78, 0.95)	0.68 (0.52, 0.88)	
Other groups	46	1	0.97 (0.93, 1)	0.97 (0.93, 1)	

^a^*CI* Confidence interval

Multivariate cox regression analysis showed that first mammogram cancer diagnoses were significantly associated with OS even after adjusting for other factors (HR 1.73, *p* = 0.04). It also revealed significant associations between triple-negative breast cancer (HR 2.85, *p* = 0.001), onset of menarche at < 12 years of age (HR 2.51, *p* = 0.009), and clinical stages II (HR 3.39, *p* = 0.006), III (HR. 12.01, *p* < 0.0001), and IV (HR 15.68, *p* < 0.0001) with OS. Age at diagnosis was not associated with OS (Table 6).

**Table 6:** Multivariate analysis of factors associated with OS

Factor	Comparison	Hazard ratio (95% confidence interval)	*P*-value
First mammogram diagnosis	Yes vs. No	1.728 (1.023, 2.92)	0.04
Subtype	HER2 Enriched vs. Luminal A	1.27 (0.502, 3.213)	0.61
	Luminal B vs. Luminal A	1.611 (0.612, 4.239)	0.33
	Luminal B-like vs. Luminal A	1.145 (0.509, 2.577)	0.74
	TNBC vs. Luminal A	2.851 (1.505, 5.399)	0.001
Age at menarche	< 12 vs. 12 and older	2.200 (1.217, 3.976)	0.009
Clinical Stage at diagnosis	Stage II vs. Stage I	3.389 (1.425, 8.061)	0.006
	Stage III vs. Stage I	12.007 (4.887, 29.498)	< 0.0001
	Stage IV vs. Stage I	15.679 (5.605, 43.859)	< 0.0001
	DCIS vs. I	0.791 (0.097, 6.452)	0.83
Age at diagnosis	1 year increase	1.149 (0.981, 1.347)	0.09

## Discussion

This study set out to determine whether women diagnosed with BC after abnormal findings on their first mammogram experience worse survival than women diagnosed with BC on subsequent mammograms. Our findings indicate compromised RFS and OS in young women with first verses subsequent mammogram cancers. Multivariate analysis revealed that the association between first mammogram cancer diagnoses and poorer RFS on Kaplan–Meier survival analysis was likely driven disease burden.

Our previous work demonstrated that women diagnosed with first mammogram cancers presented with significantly more advanced disease and were more likely to be symptomatic at presentation. This may clarify why, after adjusting for clinical stage and presence of symptoms at diagnosis, the relationship between diagnosis on first mammogram and RFS was no longer significant. The relationship between diagnosis on first mammogram and OS, however, remained significant after adjusting for all other factors. Overall, factors significantly associated with RFS were exclusive to extent of disease burden at presentation, whereas those significantly associated with OS also included patient-specific clinical history, such as age of menarche and breast cancer subtype, particularly TNBC.

While early age of menarche (age < 12 years) is a well reported risk factor for the development of breast cancer, its prognostic role is not as well discussed [17–19]. Others have previously reported an association between “very early menarche,” defined as menarche at ≤ 11 years of age, and poor breast cancer survival, with noted improvement in survival observed with incremental increase in menarchial age [20]. Another factor which showed a significant effect on OS was BC subtype. Given the inapplicability of targeted anti-hormone receptor or anti-HER2 receptor therapies which have proven to be efficacious in other breast cancer subtypes and the more aggressive biologic behavior of TNBCs, our findings of a significant association between TNBC and OS survival align with previous reports, [21–23] particularly in this young patient cohort [23].

In review of cumulative incidence of disease recurrence, we found that locoregional recurrence was relatively rare and that most recurrences in this cohort of patients were distant. This is similar to findings of other investigators such as Larsen, et al., who likewise identified higher rates of distant recurrence (15.2%) than locoregional recurrence (5.6%) in a cohort of young women with breast cancer. These investigators also found that disease stage showed the greatest effect on recurrence-free survival, even compared to tumor characteristics or surgical management, which supports our findings [24]. Similarly, a study of young women with breast cancer in Mexico found that distant metastases accounted for a majority of all noted recurrences (77%) compared to locoregional recurrences (23%) [25]. Of note, both studies reviewed young breast cancer patients < age 40 whereas our investigation focuses on the 40–45 age subgroup. Even some studies reviewing BC recurrence patterns in women ≥ 65 report a preponderance of distant metastases among total cases of recurrence [26]. Given the multitude of studies reporting overall recurrences to be more common in women diagnosed with BC at young ages, [27–29] our findings of compromised recurrence-free survival in women diagnosed with cancer on their first mammogram are clinically pertinent and reinforce the importance of screening commencement starting no later than age 40 to better enable BC detection at early stages.

Given our previous findings of significant differences in rates first mammogram cancer diagnoses by race, in which nearly half of Black women (47.6%) diagnosed with BC from ages 40–45 were diagnosed on their first mammogram compared to 26.6% of White and 34.8% of women of other races/ethnicities (*p* < 0.001), [16] we also wanted to investigate differences in oncologic outcomes by race/ethnicity in the present study. We found significantly worse OS among Black women at both 5 and 10 years after diagnosis. There was also a strong trend toward worse RFS in Black women, though no statistical significance was reached. These outcomes were not particularly surprising given that this study showed significant associations between first mammogram cancer diagnoses and both RFS and OS. Worse BC survival in Black women has also been widely reported by others, [30–32] including those specifically investigating BC in young women, in which survival disparities only worsen [30]. Though important relationships exist between race, social determinants of health, and oncologic survival, many have reported that these disparities exist even after controlling for these factors [33–36]. Some studies have emphasized the role of adverse tumor features in Black women, translating to more advanced stage at presentation [37, 38]. This may explain why race was not an independent predictor of RFS or OS on multivariate analysis after controlling for factors related to disease burden, which did show statistically significant associations with survival. Race was also not an independent predictor of OS after controlling for cancer subtype, particularly TNBC. Black women are three times more likely to have TNBCs [39] and are still more likely to die from their disease than White women with TNBC [40].

### Limitations

The retrospective and single hospital system nature of this study are important limitations. It should be noted, however, most patients from the reviewed cohort were treated at a quaternary care hospital which provides care to patients residing within various US states and international territories. Though this limits selection bias for study enrollment, some patients from distant areas pursued follow up with local oncologists after completing treatment, particularly further into their survivorship. This explains the 17.2% of patients for which recurrence status was unknown. Breast cancers diagnosed by age 45 represent roughly 10% of all BC cases in the United States; [41] thus, our inclusion criteria limited our sample size. Lastly, the demographics of patients seen within our hospital system limited diversity: Black patients represented only 11% of patients in this study and, due to insufficient numbers, other racial/ethnic minority groups could not be analyzed independently.

## Conclusion

First mammogram cancers were associated with worse survival in our study cohort, reinforcing the importance of consistent guidelines for screening commencement at age 40. Lack of cohesion in screening guidelines may disproportionately affect outcomes for groups such as Black women, who develop breast cancers at younger ages, may be more likely to have cancer detected on their first mammogram, are more likely to present with later stage disease, and have unfavorable tumor characteristics such as TNBC subtype. A multi-institutional investigation assessing the relationship between first mammogram cancer diagnoses and oncologic outcomes in a more diverse patient cohort is essential to validate our findings, as well as better clarify findings for women of other racial and ethnic minority groups.

## Data Availability

No datasets were generated or analysed during the current study.
